# A Five-Year Comparative Effectiveness Analysis of Anterior Cervical Discectomy and Fusion Versus Cervical Total Disc Replacement: A Retrospective Multiparametric Evaluation of Clinical, Radiological, and Quality of Life Outcomes

**DOI:** 10.7759/cureus.101762

**Published:** 2026-01-18

**Authors:** Bharat R Dave, Sandesh Subhash Agrawal, Arjit Vashishtha, Ajay Krishnan, Shivanand C Mayi, Ravi Ranjan Rai, Mirant B Dave, Mikeson Panthackel, Amritesh Singh, Saurabh S Kulkarni, Yogenkumar Adodariya

**Affiliations:** 1 Spine Surgery, Stavya Spine Hospital and Research Institute, Ahmedabad, IND; 2 Spine Surgery, Shree Narayana Hospital, Raipur, IND; 3 Orthopedics, Sri Devaraj Urs Medical College-Sri Devaraj URS Academy of Higher Education and Research Centre (SDUMC-SDUAHER), Kolar, IND; 4 Orthopedics and Traumatology, Shri Balaji Institute of Medical Sciences (SBIMS), Raipur, IND; 5 Spine Surgery, Bhavnagar Institute of Medical Sciences (BIMS), Bhavnagar, IND

**Keywords:** adjacent segment disease (asd), anterior cervical discectomy and fusion (acdf), anterior cervical surgery, cervical intervertebral disc degeneration, total disc replacement (tdr)

## Abstract

Background: Anterior cervical discectomy and fusion (ACDF) has long served as the gold standard for treating single-level cervical disc pathology due to its reliable decompression, restoration of cervical alignment, and high fusion rates. However, fusion-induced loss of motion may alter biomechanical loading across adjacent segments, predisposing patients to adjacent segment degeneration (ASDeg) or adjacent segment disease (ASDis), thereby affecting long-term outcomes. Total disc replacement (TDR), developed as a motion-preserving alternative, aims to maintain physiological cervical kinematics and reduce adjacent segment stress. Although both procedures demonstrate favorable short- and mid-term outcomes, comparative long-term evidence remains inconsistent. This study aimed to evaluate and compare the clinical, functional, and radiological outcomes of ACDF and TDR in patients with a comparable age distribution with single-level cervical disc prolapse or stenosis, with a minimum follow-up of five years.

Method and materials: Out of 2258 cervical spine patients operated at our institute, a retrospective cohort analysis was conducted on 73 patients with comparable age distribution who underwent surgical intervention for single-level cervical disc disease. Of these, 37 patients (50.7%) underwent TDR, and 36 patients (49.3%) underwent ACDF. Only patients with a minimum postoperative follow-up of five years were included. Functional outcomes were assessed using the visual analog scale (VAS) for neck and arm pain, Oswestry Disability Index (ODI), modified Japanese Orthopaedic Association (mJOA), and Nurick grade. Radiographic assessment included cervical range of motion (ROM) at the index, superior, and inferior adjacent segments using standardized flexion-extension radiographs. Appropriate statistical analyses were performed to determine intergroup significance.

Results: The mean follow-up duration was 5.79 ± 2.96 years in the TDR cohort and 10.88 ± 2.86 years in the ACDF cohort. The improvement in VAS for neck pain, arm pain, and ODI was not significantly different between the two groups (p = 0.479). The improvement in mJOA and Nurick grade score was seen in both the TDR and ACDF groups, but was not statistically significant (p = 0.66, p = 0.218). The absolute values of change in ROM at upper and lower adjacent levels were significantly higher (p = 0.002, p < 0.001) in the ACDF group as compared to the TDR group. The mean follow-up duration was longer in the ACDF group, reflecting earlier adoption of fusion techniques during the study period. The last follow-up radiographs have shown maintenance of cervical lordosis in TDR patients.

Conclusions: TDR and ACDF both resulted in reductions in neck and arm pain and significant improvement in disability outcomes. Both procedures provided comparable improvements in myelopathy-related functional scores. TDR was associated with preservation of cervical alignment and segmental motion, while achieving clinical and neurological outcomes comparable to ACDF.

## Introduction

Anterior cervical discectomy and fusion (ACDF) continues to be regarded as the benchmark surgical intervention for single-level cervical disc disease, particularly in patients presenting with radiculopathy, myelopathy, or combined myeloradiculopathy [1,2]. The technique involves an anterolateral cervical approach for disc excision and interbody fusion, enabling effective neural decompression, restoration of cervical lordosis, maintenance of disc height, and provision of immediate segmental stability [1,2]. Despite its proven clinical success, the obligatory segmental immobilization inherent to ACDF leads to altered cervical biomechanics and increased mechanical loading across adjacent motion segments [3,4]. This predisposes patients to adjacent segment pathology, which encompasses adjacent segment degeneration (ASDeg), radiological deterioration without clinical symptoms, and adjacent segment disease (ASDis), defined by symptomatic deterioration requiring clinical or surgical intervention [4,5].

Longitudinal clinical series have demonstrated that symptomatic ASDis develops at an estimated rate of nearly 2.9% per year, with approximately 25.6% (96/374) of patients expected to manifest new disease at an adjacent level within a decade following fusion [5,6]. Radiographic ASDeg is even more prevalent, with reported rates as high as 92% (266/288) on extended follow-up [6]. Cervical total disc replacement (TDR) has emerged as a biomechanically advantageous, motion-preserving alternative that seeks to mitigate the drawbacks associated with fusion. By maintaining near-physiological segmental kinematics, TDR reduces compensatory hypermobility and stress transfer to adjacent segments while preserving motion at the operated level [7]. Biomechanical investigations confirm that arthroplasty maintains segmental mobility and decreases adjacent-level motion compared with fusion. Clinical studies similarly report that TDR provides outcomes comparable to or better than ACDF in terms of pain relief, neurological improvement, functional recovery, and patient satisfaction, with lower secondary surgery rates reported in a prospective randomized trial of single-level cervical disc arthroplasty using the PRESTIGE® LP device (Medtronic Sofamor Danek USA, Inc., Memphis, TN, USA) compared with ACDF at long-term follow-up (10/164 (6.1%) vs 24/189 (12.7%)) [8]. Reported reoperation rates should be interpreted cautiously, as they are influenced by study design, implant characteristics, and duration of follow-up. Meta-analyses consistently demonstrate lower pooled rates of adjacent segment degeneration following TDR (7%-17%) relative to ACDF (25%-33%), although differences in symptomatic ASDis and reoperation rates remain variable, likely attributable to heterogeneity in study design, follow-up duration, patient selection, and implant characteristics.

Given these evolving insights, long-term comparative evaluation of ACDF and TDR remains clinically relevant. The present study aims to compare pain outcomes, neurological status, functional improvement, and segmental range of motion (ROM) at both index and adjacent levels in patients age-matched undergoing single-level ACDF or TDR, with a minimum follow-up duration of five years.

## Materials and methods

Study design and ethical approval

This retrospective cohort study was conducted at a single tertiary care spine center, i.e., Stavya Spine Hospital and Research Institute (SSHRI). Data were obtained from a prospectively maintained institutional surgical database and electronic medical records. The study protocol was approved by the Institutional Ethics Committee of SSHRI (protocol code: SSHRI/CS/NS/ACDF TDR/BRD/97/12.25) and the Clinical Trials Registry-India (CTRI/2025/12/099013). All patient identifiers were removed to ensure confidentiality. During the study period, i.e., between January 2009 and December 2020, 2258 cervical spine surgeries were performed. Patients aged 20-60 years with symptomatic, single-level cervical disc prolapse or stenosis (C3-C7), who had failed at least six weeks of conservative management and subsequently underwent either TDR or ACDF, were considered eligible. Exclusion criteria included patients who exhibited signs of heterotopic ossification during follow-up, revision procedures, local or systemic infections, neoplastic conditions, multilevel disease, or incomplete clinical or radiological records. Patients who developed advanced heterotopic ossification (McAfee grade III or IV) at final follow-up were excluded, as these cases no longer represent functional motion-preserving arthroplasty. Only patients with comparable age distribution between the ACDF and TDR groups and a minimum follow-up of two years were included. Patients with ≥5 years of follow-up were analyzed as a long-term outcome subgroup. The longer mean follow-up in the ACDF group reflects the earlier adoption of ACDF at our institute, whereas TDR was introduced later during the study period, resulting in comparatively shorter but still adequate follow-up duration. Outcome comparisons were based on change scores from baseline to final follow-up, thereby minimizing the impact of follow-up duration variability between groups.

Surgical procedures

All procedures were performed under general anaesthesia using the standard Smith-Robinson anterior cervical approach [9].

ACDF Group

The target level was verified fluoroscopically. Casper pins were placed in the vertebrae adjacent to the affected disc. Distraction was done. Discectomy was done with curettes and Kerrison rongeurs, and decompression was achieved. Partial resection of the anterior superior vertebral lip was performed, followed by the insertion of Casper distractor pins. The endplates were prepared using multiple perforations to promote fusion. Interbody fusion was achieved with an autograft-packed cage, stabilized using either a Zero-P stand-alone spacer or an anterior cervical plate construct.

TDR Group

After fluoroscopic level confirmation, discectomy and decompression were done similarly to ACDF after distracting the disc space with Casper pins. Endplate preparation was completed as per standard arthroplasty protocol. The PRESTIGE® LP prosthesis (Medtronic, USA) was sized appropriately, positioned centrally, and implanted under fluoroscopic guidance. Hemostasis was secured, thorough irrigation was done with saline, and the wound was closed in layers over a negative suction drain.

Outcome measures

Demographic and Clinical Data

Age, sex, affected disc level, and surgical indication were recorded. A detailed history and physical examination were performed preoperatively.

Radiological Assessment

Preoperative and final follow-up imaging included anteroposterior, flexion, and extension radiographs, as well as MRI scans. Sagittal plane angular motion was measured using Cobb’s technique on dynamic lateral radiographs. ROM at the operated level, one level above, and one level below was measured using Surgimap Spine software (Nemaris Inc., New York, NY). Postoperative complications such as persistent neck pain, radiculopathy, graft site infection, adjacent segment disease, and requirement for revision surgery were noted during follow-up.

Functional Assessment

Functional outcomes were assessed preoperatively and at final follow-up using the following standardized instruments:

Neck Disability Index (NDI) and Oswestry Disability Index (ODI) [10]: Patients completed standardized questionnaires assessing neck- or spine-related disability. Scores range from 0 to 100, with higher scores indicating greater disability. ODI scores were interpreted as minimal (0%-20%), moderate (21%-40%), severe (41%-60%), crippled (61%-80%), and bedbound (81%-100%). The ODI was used to assess spine-related functional impairment; higher scores reflect greater disability [10]. 

Visual analog scale (VAS) [11] for neck and arm pain: Patients marked pain intensity on a 10 cm line, where 0 = no pain and 10 = worst imaginable pain. Scores were interpreted as mild (1-3), moderate (4-6), and severe (7-10).

Modified Japanese Orthopaedic Association (mJOA) score [12]: Neurological function due to myelopathy was graded from 0 to 18, with higher scores indicating better functional status. Scores were classified as severe (0-11), moderate (12-14), and mild (15-18) myelopathy.

Nurick grade [13]: Gait dysfunction and motor impairment were graded from 0 (signs of root involvement without gait difficulty) to 5 (confined to wheelchair or bedridden), with higher grades indicating greater disability.

Clinical outcome scores were obtained from medical records, and radiological assessments were independently performed by two spine surgeons who were not involved in the index surgery and were blinded to the surgical procedure. 

Statistical analysis

Data analysis was performed using IBM SPSS Statistics for Windows, Version 25 (Released 2017; IBM Corp., Armonk, New York, United States). VAS scores (VAS-neck pain and VAS-arm pain), mJOA scores, NDI scores, Nurick grade, and ROM (at operated level, one level above, and below) were analyzed, and results were expressed as mean with standard deviations. A paired t-test was used to compare preoperative and postoperative scores. Cases with incomplete baseline or follow-up clinical and radiological data were excluded from the final analysis. No data imputation was performed. Intergroup comparisons of improvement scores between the TDR and ACDF cohorts were performed using an independent-samples t-test. Although the NDI is commonly used for cervical spine disorders, the ODI was employed in this study to maintain uniform assessment of spine-related disability across institutional protocols at the time of data collection. Baseline demographic variables, including age, were compared between groups to confirm comparability. A p-value < 0.05 was considered statistically significant.

## Results

Demographic data and symptomatology

A total of 37 patients of the TDR group and 36 patients of the ACDF group were enrolled in the study. The average age of patients in the TDR group was 39.95 ± 6.69, and in the ACDF group was 40.5 ± 5.31. There were 27 males (72.9%) and 10 females (27.02%) in the TDR group, whereas the ACDF group had 34 males (94.4%) and two females (5.5%). C5-6 level (47.94%) was the most operated in both groups, followed by C4-5 (26.02%), and C6-7 (26.02%). A total of 15 patients, each of TDR (40.54%) and ACDF (41.66%), presented with radiculopathy, 14 patients of TDR (37.83%) and 12 patients of ACDF (33.33%) presented with myeloradiculopathy, and eight patients with TDR (21.62%), and nine patients with ACDF (25%) presented with myelopathy as presenting complaints. Neck pain was present in the majority of the patients (TDR (n = 29/37) and ACDF (n = 30/36)). Similarly, radiculopathy was present in the majority of the patients (TDR (n = 27/37) and ACDF (n = 27/36)). In TDR patients, right-sided radiculopathy was predominant (n = 18), followed by no radiculopathy (n = 9), left-sided radiculopathy (n = 7), and bilateral radiculopathy (n = 3). In ACDF patients, left-sided radiculopathy was predominant (n = 13), followed by bilateral radiculopathy (n = 8), no radiculopathy (n = 8), and left-sided radiculopathy (n = 7). The mean follow-up duration was 5.79 ± 2.96 years (range: ≥5 years) in the TDR group and 10.88 ± 2.86 years in the ACDF group. Both groups were comparable in baseline characteristics except for sex distribution, with 27% females in the TDR group versus 5.6% in the ACDF group (Table 1). The longer follow-up duration in the ACDF cohort reflects earlier adoption of fusion techniques during the study period and was accounted for during the interpretation of long-term complications.

**Table 1:** Patient demographics and symptomatology

Variable	TDR (n = 37)	ACDF (n = 36)	Total (N = 73)
Age (years), mean ± SD	39.95 ± 6.69	40.5 ± 5.31	-
Sex			
Male	27 (73.0%)	34 (94.4%)	61 (83.6%)
Female	10 (27.0%)	2 (5.6%)	12 (16.4%)
Level of surgery			
C4-5	11 (29.7%)	8 (22.2%)	19 (26.0%)
C5-6	16 (43.2%)	19 (52.8%)	35 (47.9%)
C6-7	10 (27.0%)	9 (25.0%)	19 (26.0%)
Clinical presentation			
Radiculopathy	15 (40.5%)	15 (41.7%)	30 (41.1%)
Myelopathy	8 (21.6%)	9 (25.0%)	17 (23.3%)
Myeloradiculopathy	14 (37.8%)	12 (33.3%)	26 (35.6%)
Neck pain			
Absent	8 (21.6%)	6 (16.7%)	14 (19.2%)
Present	29 (78.4%)	30 (83.3%)	59 (80.8%)
Radiculopathy			
Absent	10 (27.0%)	9 (25.0%)	19 (26.0%)
Present	27 (73.0%)	27 (75.0%)	54 (74.0%)
Side of symptoms			
Absent	9 (24.3%)	8 (22.2%)	17 (23.3%)
Bilateral	3 (8.1%)	8 (22.2%)	11 (15.1%)
Left	7 (18.9%)	13 (36.1%)	20 (27.4%)
Right	18 (48.6%)	7 (19.4%)	25 (34.2%)
Average follow-up (years), mean ± SD (range)	5.79 ± 2.96 (5.0-11.75)	10.88 ± 2.86 (3.83-13.75)	-

Preoperative and postoperative functional score assessment in TDR patients

The mean preoperative VAS-neck pain was 5.62 ± 2.8, which was reduced to 1.22 ± 1.2 postoperatively and was statistically significant (p < 0.001). The mean preoperative VAS-arm pain was 8.49 ± 3, which was reduced to 1.38 ± 1.82 postoperatively and was statistically significant (p < 0.001). The mean preoperative mJOA was 14.86 ± 2.36, which improved to 17.54 ± 0.99 postoperatively and was statistically significant (p < 0.001). The mean preoperative ODI was 60.97 ± 24.82, which was reduced to 11.30 ± 11.2. Absolute change (I) is the change in ROM following the procedure, either TDR or ACDF. Absolute change in ROM (I) was calculated by subtracting the postoperative ROM from the preoperative ROM, and only the absolute values were considered. It was calculated for each operated and adjacent levels in both groups separately, and the values of the TDR and ACDF groups were compared. Change in the ROM at the operative site was significantly (p < 0.001) less in the TDR group (2.17 ± 2.24) as compared to the ACDF group (4.9 ± 3.35), implying that restoration of ROM was near preoperative ROM in the TDR group as compared to the ACDF group, which is a fusion technique. The I values of the upper adjacent level were significantly higher (p = 0.002) in the ACDF group (1.32 ± 1.12) as compared to the TDR group (0.62 ± 0.68). Similarly, the values of the lower adjacent level were significantly higher (p < 0.001) in the ACDF group (0.92 ± 0.7) as compared to the TDR group (0.30 ± 0.46). This would imply that post-ACDF, there was an increase in the ROM of both adjacent upper and lower levels as compared to the change in ROM at adjacent levels in the TDR group, and this was statistically significant (p < 0.001). The mean preoperative Nurick grade was 1.65 ± 0.89, which improved to 0.62 ± 0.79 postoperatively and was statistically significant (p < 0.001) as illustrated in Table 2.

**Table 2:** Preoperative and postoperative functional score assessment in TDR patients VAS: visual analog scale; mJOA: modified Japanese Orthopaedic Association; ODI: Oswestry Disability Index; Asymp. Sig. (two-tailed): asymptotic significance (two-tailed p-value); N: number of patients Data are presented as mean ± standard deviation (SD). Statistical analysis was performed using the paired samples t-test. p-values < 0.05 were considered statistically significant

Parameter	N	Mean ± SD (preoperative)	Mean ± SD (postoperative)	p-value (two-tailed)
VAS (neck)	37	5.62 ± 2.80	1.22 ± 1.20	<0.001
VAS (arm)	37	8.49 ± 3.00	1.38 ± 1.82	<0.001
mJOA	37	14.86 ± 2.36	17.54 ± 0.99	<0.001
ODI	37	60.97 ± 24.82	11.30 ± 11.21	<0.001
Nurick grade	37	1.65 ± 0.89	0.62 ± 0.79	<0.001

Preoperative and postoperative functional score assessment in ACDF patients

The mean preoperative VAS-neck pain was 5.47 ± 1.98, which was reduced to 2.47 ± 1.18 postoperatively and was statistically significant (p < 0.001). The mean preoperative VAS-arm pain was 6.83 ± 3.09, which was reduced to 2.27 ± 1.66 postoperatively and was statistically significant (p < 0.001). The mean preoperative mJOA was 12.88 ± 3, which improved to 15.38 ± 2.19 postoperatively and was statistically significant (p < 0.001). The mean preoperative ODI was 61.61 ± 18.02, which was reduced to 26.5 ± 9.8 postoperatively and was statistically significant (p < 0.001). The mean preoperative Nurick grade was 2.75 ± 1.2, which improved to 1.5 ± 0.81 postoperatively and was statistically significant (p < 0.001) as described in Table 3.

**Table 3:** Preoperative and postoperative functional score assessment in ACDF patients VAS: visual analog scale; mJOA: modified Japanese Orthopaedic Association; ODI: Oswestry Disability Index; Asymp. Sig. (two-tailed): asymptotic significance (two-tailed p-value); N: number of patients Data are presented as mean ± standard deviation (SD). Statistical analysis was performed using the paired samples t-test. p-values < 0.05 were considered statistically significant

Parameter	N	Preoperative (mean ± SD)	Postoperative (mean ± SD)	p-value (two-tailed)
VAS (neck)	36	5.47 ± 1.98	2.47 ± 1.18	<0.001
VAS (arm)	36	6.83 ± 3.09	2.27 ± 1.66	<0.001
mJOA	36	12.88 ± 3	15.38 ± 2.19	<0.001
ODI	36	61.61 ± 18.02	26.50 ± 9.80	<0.001
Nurick grade	36	2.75 ± 1.20	1.50 ± 0.81	<0.001

Preoperative and postoperative radiological score assessment in TDR patients

The mean preoperative ROM at the operative site was 4.84 ± 2.07°, which increased to 5.20 ± 2.97° postoperatively. This change was not statistically significant (t = 0.72, df = 36, mean difference 0.36°; 95% CI: -0.46 to 1.18; p = 0.479). The mean ROM at the adjacent upper level increased from 0.41 ± 0.59° preoperatively to 1.03 ± 0.69° postoperatively, which was statistically significant (t = 5.84, df = 36, mean difference 0.62°; 95% CI: 0.41-0.83; p < 0.001). The mean ROM at the adjacent lower level increased from 0.46 ± 0.61° to 0.70 ± 0.52°, which was also statistically significant (t = 3.28, df = 36, mean difference 0.24°; 95% CI: 0.07-0.41; p = 0.002) (Table 4). These findings indicate that TDR preserves ROM at the operative site, with compensatory increases at the adjacent levels.

**Table 4:** Preoperative and postoperative radiological score assessment in TDR patients ROM : range of motion; °: degrees; df: degrees of freedom; 95% CI: 95% confidence interval of the mean difference Data are expressed as mean ± standard deviation (SD). Statistical analysis was performed using a paired samples t-test. p-values < 0.05 were considered statistically significant

Parameter	N	Preoperative (mean ± SD)	Postoperative (mean ± SD)	t-value	df	p-value	Mean difference (post–pre)
ROM at operative site (°)	37	4.84 ± 2.07	5.20 ± 2.97	0.72	36	0.479	0.36
Adjacent upper-level ROM (°)	37	0.41 ± 0.59	1.03 ± 0.69	5.84	36	<0.001	0.62
Adjacent lower-level ROM (°)	37	0.46 ± 0.61	0.70 ± 0.52	3.28	36	0.002	0.24

Preoperative and postoperative radiological score assessment in ACDF patients

The mean preoperative ROM at the operative site was 5.22 ± 3.31, which decreased to 0.32 ± 0.28 postoperatively and was statistically significant, indicating successful fusion at the operative level (t = 9.56, p < 0.001). The mean ROM at the adjacent upper level preoperatively was 2.72 ± 1.95, which increased slightly to 2.81 ± 2.34 postoperatively but was not statistically significant (t = 0.29, p = 0.776). The mean ROM at the adjacent lower level preoperatively was 1.34 ± 1.24, which decreased to 1.23 ± 0.87 postoperatively and was also not statistically significant (t = 0.59, p = 0.560) (Table 5).

**Table 5:** Preoperative and postoperative radiological score assessment in ACDF patients ROM: range of motion; °: degrees; df: degrees of freedom; 95% CI: 95% confidence interval of the mean difference Data are expressed as mean ± standard deviation (SD). Statistical analysis was performed using a paired samples t-test. p-values < 0.05 were considered statistically significant

Parameter	N	Preoperative (mean ± SD)	Postoperative (mean ± SD)	t-value	df	p-value	Mean difference (post-pre)
ROM at operative Site (°)	36	5.22 ± 3.31	0.32 ± 0.28	9.56	35	<0.001	-4.90
Adjacent upper-level ROM (°)	36	2.72 ± 1.95	2.81 ± 2.34	0.29	35	0.776	0.09
Adjacent lower-level ROM (°)	36	1.34 ± 1.24	1.23 ± 0.87	0.59	35	0.560	-0.11

Comparison of the difference (D) in functional scores between TDR and ACDF patients

D is the improvement following the procedure, either TDR or ACDF. D was calculated by subtracting the postoperative scores from the preoperative scores, and only the absolute values were considered. D was calculated for each parameter in both groups separately, and the values of the TDR and ACDF groups were compared. The improvement in neck pain was significantly greater (p = 0.007) in the TDR group (4.41 ± 2.71) as compared to the ACDF group (3.00 ± 1.33). The improvement in arm pain was significantly greater (p < 0.001) in the TDR group (7.11 ± 3.23) as compared to the ACDF group (4.56 ± 2.45). The improvement in ODI was significantly greater (p = 0.003) in the TDR group (49.89 ± 23.87) as compared to the ACDF group (35.11 ± 15.46). The improvement in mJOA score was seen in both the TDR (2.68 ± 2.11) and ACDF (2.5 ± 1.13) groups, but was not statistically significant (p = 0.66). The improvement in Nurick grade was seen in both the TDR (1.03 ± 0.80) and ACDF (1.25± 0.73) groups, but was not statistically significant (p = 0.218) as shown in Table 6.

**Table 6:** Comparison of difference of preoperative and postoperative functional scores (D) VAS: visual analog scale; mJOA: modified Japanese Orthopaedic Association; ODI: Oswestry Disability Index; TDR: total disc replacement; ACDF: anterior cervical discectomy and fusion Comparison of differences (D) represents the change between preoperative and postoperative scores for each parameter. Data are expressed as mean ± standard deviation (SD). Statistical analysis was performed using the independent samples t-test. p-values < 0.05 were considered statistically significant

Parameter	N (TDR)	N (ACDF)	Mean ± SD (TDR)	Mean ± SD (ACDF)	p-value (two-tailed)
Improvement in VAS (neck)	37	36	4.41 ± 2.71	3.00 ± 1.33	0.007
Improvement in VAS (arm)	37	36	7.11 ± 3.23	4.56 ± 2.45	<0.001
Improvement in mJOA	37	36	2.68 ±2.11	2.5 ± 1.13	0.660
Improvement in ODI	37	36	49.89 ± 23.87	35.11 ± 15.46	0.003
Improvement in Nurick grade	37	36	1.03 ± 0.80	1.25 ± 0.73	0.218

Comparison of absolute change in ROM (I) between TDR and ACDF patients

Absolute change (I) is the change in ROM following the procedure, either TDR or ACDF. Absolute change in ROM (I) was calculated by subtracting the postoperative ROM from the preoperative ROM, and only the absolute values were considered. It was calculated for each operated and adjacent levels in both groups separately, and the values of the TDR and ACDF groups were compared. Change in the ROM at the operative site was significantly (p < 0.001) less in the TDR group (2.17 ± 2.24) as compared to the ACDF group (4.9 ± 3.35), implying that restoration of ROM was near preoperative ROM in the TDR group as compared to the ACDF group, which is a fusion technique. The I values of the upper adjacent level were significantly higher (p = 0.002) in the ACDF group (1.32 ± 1.12) as compared to the TDR group (0.62 ± 0.68). Similarly, the values of the lower adjacent level were significantly higher (p < 0.001) in the ACDF group (0.92 ± 0.7) as compared to the TDR group (0.30 ± 0.46). Although absolute ROM change at adjacent levels was numerically higher in the ACDF group, this reflects compensatory hypermobility rather than physiological motion, whereas TDR preserved controlled segmental kinematics (Table 7).

**Table 7:** Absolute change in preoperative and postoperative ROM (I) ROM: range of motion; °: degrees; TDR: total disc replacement; ACDF: anterior cervical discectomy and fusion Absolute change (I*) represents the difference between preoperative and postoperative ROM values at each spinal level. Data are presented as mean ± standard deviation (SD). Statistical analysis was performed using the independent samples t-test. p-values < 0.05 were considered statistically significant

Parameter	N (TDR)	N (ACDF)	Mean ± SD (TDR)	Mean ± SD (ACDF)
Change in ROM at operative site (°)	37	36	2.17 ± 2.24	4.90 ± 3.35
Change in adjacent upper-level ROM (°)	37	36	0.62 ± 0.68	1.32 ± 1.12
Change in adjacent lower-level ROM (°)	37	36	0.30 ± 0.46	0.92 ± 0.70

Comparison of mean cervical lordosis and ROM between ACDF and TDR

The mean overall cervical spine lordosis (C2-C7 lordosis) in flexion and extension at the last follow-up was found to be -19.63 ± 10.43 and 25.74 ± 13.28°, respectively, in the TDR group, as compared to -16.24 ± 9.56 and 21.63 ± 12.74 in the ACDF group. The mean overall cervical spine ROM was found to be 37.59 ± 20.25° in the TDR group as compared to 24.93 ± 16.34 in the ACDF group (negative value represents kyphosis) (Table 8).

**Table 8:** Comparison of cervical spine lordosis in flexion and extension and overall cervical spine ROM between the TDR and the ACDF groups ROM: range of motion; °: degrees; TDR: total disc replacement; ACDF: anterior cervical discectomy and fusion Data are presented as mean ± standard deviation (SD). Negative values represent kyphosis

Parameter	TDR	ACDF
C2-C7 lordosis in flexion (°)	-19.63 ± 10.43	-16.24 ± 9.56
C2-C7 lordosis in extension(°)	25.74 ± 13.28	21.63 ± 12.74
Overall cervical C2-C7 ROM(°)	37.59 ± 20.25	24.93 ± 16.34

Postoperative complications and residual symptoms

ASDis was noted in 7/36 patients (19.44%) in the ACDF group and was statistically greater (p = 0.019) than the TDR group (0%). Out of the seven patients with ASDis, five (71.42%) had lower-level ASDis, and two (28.57%) had upper-level ASDis. Five patients (13.88%) in the ACDF group were advised reoperation for ASDis, which was statistically greater (p = 0.019) than the TDR group (0%). The remaining two patients with ASDis in the ACDF group were planned to be managed conservatively.

Five patients (13.88%) in the ACDF group and one patient (2.7%) in the TDR group had residual neck pain and were comparable in both groups (p = 0.082). Three patients each in both groups complained of trapezius pain. The postoperative complications and residual symptoms are summarised in Table 9. Interpretation of adjacent segment disease and reoperation rates should consider the longer follow-up duration in the ACDF group, which may increase the observed incidence of time-dependent complications.

**Table 9:** Postoperative complications and residual symptoms TDR: total disc replacement; ACDF: anterior cervical discectomy and fusion; B/L: bilateral; LT: left side; RT: right side

Parameter/complication	TDR (n, %)	ACDF (n, %)	Total (n)
Adjacent segment degeneration			
Upper level	0	2 (5.55%)	2
Lower level	0	5 (13.8%)	5
NIL	37 (100%)	29 (80.55%)	66
Adjacent segment disease			
Managed conservatively	0	2 (5.55%)	2
Revision surgery advised	0	5 (13.8%)	5
Neck pain			
Absent	36 (97.3%)	31 (86.1%)	67
Present	1 (2.7%)	5 (13.9%)	6
Trapezius pain			
Bilateral	2 (5.4%)	2 (5.5%)	4
Left	1 (2.7%)	0	1
Right	0	1 (2.7%)	1
Absent	34 (91.8%)	33 (91.6%)	67

## Discussion

ACDF has been the treatment of choice for symptomatic single-level cervical disc prolapse/stenosis with cervical radiculopathy/myeloradiculopathy. Fusion of motion segments leads to increased intradiscal pressure in adjacent segments. Studies with longer follow-up have suggested that >25% patients develop symptomatic ASDis within 10 years after ACDF [14]. In properly selected patients, TDR is a potential alternative following decompression. TDR preserves motion at the index segment and limits the adjacent segment stresses and degeneration.

Demographic assessment

The majority of studies comparing ACDF and TDR with a minimum follow-up of five years reported the average age of patients in the TDR group to be between 42.1 and 44.4 years, and in the ACDF group, 43-44.7 years [15-19]. We had a similar age distribution in our study, with the average age of patients in the TDR group being 39.95 years, and in the ACDF group being 40.5 years (Table 10).

**Table 10:** Demographic characteristics and study design of patients undergoing TDR and ACDF across comparative studies NS: not significant; N/A: data not available; RCT: randomized controlled trial; TDR: total disc replacement; ACDF: anterior cervical discectomy and fusion Values are expressed as mean ± standard deviation unless otherwise stated. The p-values from t-tests compare the mean age between the TDR and ACDF groups. The p-values from the Chi-square (χ²) tests compare the gender distribution between the TDR and ACDF groups

SL no	Study (reference)	Study design	Sample size (TDR)	Sample size (ACDF)	Mean age (years, TDR)	Mean age (Years, ACDF)	Gender (M/F, TDR)	Gender (M/F, ACDF)	Follow-up (years, TDR)	Follow-up (years, ACDF)	Statistical test (t)	p-value (age, t-test)	Statistical test (χ²)	p-value (gender, χ² test)
1	Porchet et al. [14]	Prospective RCT	27	28	44 ± 8.9	43 ± 6.9	17/10	12/16	2	2	t = 0.35	0.73	χ² = 1.21	NS
2	Murrey et al. [15]	Prospective RCT	103	106	42.1 ± 8.4	43.5 ± 7.1	46/57	49/57	2	2	t = 1.12	0.26	χ² = 0.21	NS
3	Garrido et al. [17]	Prospective cohort	21	26	44	N/A	23/18	N/A	3	3	–	–	–	–
4	Burkus et al. [18]	Prospective RCT	276	265	43.3	43.9	128/148	122/143	5	5	t = 0.63	0.53	χ² = 0.54	NS
5	Sasso et al.[19]	Prospective RCT	242	221	44.4 (25–78)	44.7 (27–68)	110/132	113/108	4	4	–	–	χ² = 0.12	NS
6	Coric et al. [20]	Prospective RCT	136	133	43.7 ± 7.76	43.9 ± 7.39	51/85	59/74	2	2	t = 0.17	0.86	χ² = 2.16	NS
7	Jawahar et al. [21]	Prospective cohort	59	34	N/A	N/A	21/38	16/18	2	2	–	–	χ² = 0.03	NS
8	Nabhan et al. [22]	Prospective cohort	20	21	44	N/A	23/18	N/A	3	3	–	–	–	–
9	Present study	Retrospective cohort	37	36	39.95 ± 6.69	40.5 ± 5.31	27/10	34/2	5.79	10.88	t = 0.47	0.64	χ² = 16.42	<0.001

Functional score assessment

The VAS neck pain improved significantly at final follow-up in both groups from preoperative scores. The absolute improvement in TDR (4.41 ± 2.71/10) was significantly (p < 0.007) higher than the absolute improvement in the ACDF group (3.00 ± 1.33/10). Similar to our results, Garrido et al. reported improvement of 82% in the TDR group as compared to 67% in the ACDF group [19-22]. Most of the studies report an absolute improvement between 46 and 62.6/100 points in the TDR group and between 43 and 61.6/100 points in ACDF, with similar improvement in both groups. VAS arm pain improved significantly at final follow-up in both groups from preoperative scores, and absolute improvement in the TDR group (7.11 ± 3.23) was significantly (p < 0.001) higher than absolute improvement in the ACDF group (4.56 ± 2.45). Similar to our results, Garrido et al. reported improvement of 86% in arm pain in the TDR group as compared to 73% in the ACDF group. Most of the studies report improvement in the range of 52.5-68 points in the TDR group and between 47.7 and 55.4 points in the ACDF, with similar improvement in both groups as described in Table 11.

**Table 11:** Comparison of preoperative and final follow-up functional outcomes between the TDR and ACDF groups VAS: visual analog scale; ODI: Oswestry Disability Index; NDI: Neck Disability Index; NR: not reported; NS: not significant An independent-samples t-test was used to compare continuous functional outcomes (VAS, ODI, NDI, Nurick grade) between TDR and ACDF groups, while paired t-tests were used for within-group pre- and postoperative comparisons. The p-values indicate comparison between the TDR and ACDF groups at the final follow-up. The p-value represents the comparison of postoperative NDI scores between the TDR and ACDF groups

Study	VAS neck pre (TDR)	VAS neck post (TDR)	VAS arm pre (TDR)	VAS arm post (TDR)	Disability measure	Pre (TDR)	Post (TDR)	Pre (ACDF)	Post (ACDF)	p-value (postoperative: TDR vs ACDF)
Porchet et al. [14]	N/A	Improved	N/A	Improved	NDI	N/A	Improved	N/A	N/A	N/A
Murrey et al. [15]	42.1	46	43	44	NDI	53.9	21.4	N/A	N/A	N/A
Garrido et al. [17]	76.2	13.6	78.8	10.8	NDI	51.1	10.1	N/A	N/A	N/A
Burkus et al. [18]	68.2	12.2	59.1	6.6	NDI	55.7	17.3	N/A	N/A	N/A
Sasso et al. [19]	75.4	20.7	71.2	16.6	NDI	13.2	19.8	N/A	N/A	N/A
Coric et al. [20]	77.1	23.6	N/A	N/A	NDI	63.2	22.6	N/A	N/A	N/A
Jawahar et al. [21]	N/A	N/A	N/A	N/A	NDI	44.9	43	N/A	N/A	N/A
Our study	56.2	12.2	84.9	13.8	ODI	60.97	11.3	59.2	24.1	< 0.001

ODI scores improved significantly at final follow-up in both groups from preoperative scores; however, the absolute improvement in TDR (49.89 ± 23.87) was significantly (p < 0.003) higher than the absolute improvement in the ACDF group (35.11 ± 15.46). Similar to our results, Garrido et al. reported an NDI success of 93.3% in the TDR group as compared to 82.4% in the ACDF group. Long-term studies in the literature report absolute improvement of ODI between 38.4 and 44.9 in the TDR group and 34.1 and 43 in the ACDF group, with similar improvements in both groups (Table 10). Myelopathy assessment scores, like mJOA and Nurick grades, were included. The mJOA scores improved significantly at final follow-up in both groups from preoperative scores. The absolute improvement in the TDR group (2.68 ± 2.11) was statistically similar (p = 0.66) to the ACDF group (2.5 ± 1.13). Nurick grade improved significantly at final follow-up in both groups from the preoperative grade. The absolute improvement in the TDR group (1.03 ± 0.8) was statistically similar (p = 0.218) to the ACDF group (1.25 ± 0.73). None of the studies comparing ACDF and TDR outcomes included mJOA and Nurick grade. TDR group performed better in terms of scores assessing neck pain, arm pain, and disability due to neck pain, and similar in terms of scores assessing myelopathy, like mJOA and Nurick grade, when compared to the ACDF group.

Preoperative and postoperative disability outcomes for our cohort are summarized in Table 11. In patients undergoing TDR, the mean ODI decreased from 60.97 ± 12.4 preoperatively to 11.3 ± 6.7 postoperatively, representing a statistically significant improvement (p < 0.001). In the ACDF group, the mean ODI decreased from 59.2 ± 13.1 preoperatively to 24.1 ± 10.8 postoperatively. Comparison of postoperative ODI between TDR and ACDF demonstrated significantly greater improvement in the TDR group (p < 0.001). For clarity, other published studies in Table 11 report disability using the NDI where applicable, while our study consistently reports ODI.

Radiological score assessment

ROM at index level was increased (5.2 ± 2.97 d°) at final follow-up as compared to preoperative ROM (4.84 ± 2.07 d°) in TDR group but was not statistically significant (p = 0.479), whereas ROM at index level significantly (p = 0.001) decreased at final follow-up (0.32 ± 0.28°) as compared to preoperative ROM (5.22 ± 3.31°) in ACDF group, implying fusion at the operated level. Similarly, Sasso et al. reported an increase in ROM at the index level at the final follow-up (8.5°) as compared to preoperative levels (6.5°) in the TDR group [19]. Most of the studies reported absolute change between 1.6 and 9.36° for the TDR group and 5.2 and 7.3° for the ACDF group. In our study, the absolute change was 0.36° for the TDR group and 4.9° in the ACDF group. This is suggestive of maintenance/improvement of ROM at the index level in the TDR group and abolition of movement at the index level in the ACDF group.

Evaluation of ROM at adjacent levels within each group (ACDF and TDR) shows that ROM at adjacent upper level significantly (p < 0.001) increased at the final follow-up (1.03 ± 0.69) as compared to the preoperative level (0.41 ± 0.59) in TDR group, whereas ROM at the adjacent upper level at the final follow-up (2.81 ± 2.34) was similar (p = 0.776) to the preoperative ROM (2.72 ± 1.95) in the ACDF group. ROM at the adjacent lower level significantly (p = 0.007) increased at the final follow-up (0.7 ± 0.52) as compared to the preoperative level (0.46 ± 0.61) in the TDR group, whereas ROM at the adjacent lower level at the final follow-up (1.23 ± 0.87) was similar (p = 0.56) to the preoperative ROM (1.34 ± 1.24) in the ACDF group. However, the absolute change in ROM at adjacent levels was found to be greater in the TDR group as compared to the ACDF group. None of the studies comparing long-term results of ACDF and TDR reported ROM at adjacent upper and lower levels. The TDR group had better restoration of ROM at the index level, adjacent upper and lower levels, as compared to the ACDF group. This restoration of ROM and improved cervical kinematics can lead to decreased stresses at adjacent levels and prevent/halt further degeneration of adjacent segments (Table 12). Although absolute ROM change at adjacent levels was numerically higher in the ACDF group, this reflects compensatory hypermobility rather than physiological motion, whereas TDR preserved controlled segmental kinematics.

**Table 12:** Comparison of cervical ROM between the TDR and ACDF groups at index and adjacent levels ROM: range of motion; TDR: total disc replacement; ACDF: anterior cervical discectomy and fusion; NR: not reported; NS: not significant An independent-samples t-test was used to compare ROM between TDR and ACDF groups, while paired t-tests were used for within-group comparisons. The p-values represent intergroup comparisons at final follow-up. The p-values represent comparisons of the change in ROM between the TDR and ACDF groups at the index and adjacent levels

Level	Study	Preop ROM (TDR)	Final ROM (TDR)	Change (TDR)	Preop ROM (ACDF)	Final ROM (ACDF)	Change (ACDF)	p-value (change in ROM: TDR vs ACDF)	Statistical test
Index	Porchet et al. [14]	5.9	5.9	0	6.3	1.1	5.2	N/A	N/A
Index	Sasso et al. [19]	6.5	8.5	2	8.4	1.1	7.3	N/A	N/A
Index	Coric et al. [20]	8.2	9.8	1.6	7.6	0.8	6.8	N/A	N/A
Index	Our study	4.84	5.2	0.36	5.22	0.32	4.9	< 0.001	t-test
Adjacent upper level	Our study	0.41	1.03	0.62	2.72	2.81	0.09	0.02	t-test
Adjacent lower level	Our study	0.46	0.7	0.24	1.34	1.23	0.11	0.04	t-test

The mean overall cervical spine lordosis (C2-C7 lordosis) at last follow-up was found to be 37.59 ± 20.25° in the TDR group as compared to 24.93 ± 16.34 in the ACDF group. Owing to the preserved mobility at the operated level and transferring minimal stresses to the adjacent segments, the overall cervical spine alignment and kinematics are found to be maintained in patients who underwent TDR as compared to ACDF. To our knowledge, this is among the few long-term comparative studies evaluating myelopathy-specific outcomes using mJOA and Nurick grading following TDR versus ACDF, demonstrating comparable neurological recovery with both techniques.

Assessment of postoperative complications and residual symptoms

The reported incidence of ASDeg and ASDis in ACDF (4.07-13.88%) is higher than that of TDR (3.98-6.61%) (Table 13). We had similar results with the incidence of ASDis in the ACDF group (19.44%), which was statistically greater (p = 0.019) than in the TDR group (0%). Out of the seven patients with ASDis, five patients (71.42%) had lower-level ASDis, and two patients (28.57%) had upper-level ASDis. Five patients (13.88%) in the ACDF group were advised reoperation for ASDis, which was statistically greater (p = 0.019) than that in the TDR group (0%). The remaining two patients with ASDis in the ACDF group were planned to be managed conservatively. The TDR group with better restoration of cervical kinematics led to decreased stresses at the adjacent level and decreased incidence of ASDeg and ASDis. A small proportion of patients postoperatively complain of persistent neck pain. The reported incidence of persistent neck pain in the ACDF group (2.26, 50%) is greater than in the TDR group (1.23, 25.9%). We had similar results with persistent neck pain common in the ACDF group (13.8%) as compared to the TDR group (2.7%). Incidence of trapezius pain was similar in both groups (three patients each).

**Table 13:** Postoperative complications, residual symptoms, and adjacent segment disease following TDR and ACDF TDR: total disc replacement; ACDF: anterior cervical discectomy and fusion; N/A: not reported

Outcome	Study	TDR	ACDF
Graft site infection	Porchet et al. [14] to Nabhan et al. [22]	N/A	N/A
	Our study	Nil	3
Other complications	Porchet et al. [14]	1 (pancreatitis)	N/A
	Murrey et al. [15]	4 (continued pain, dural tear, implant removal/conversion)	7 (pseudoarthrosis, plate subsidence, dysphagia, wound infection, foraminotomy)
	Garrido et al. [17]	N/A	2 (pseudoarthrosis, nonadjacent level disease)
	Burkus et al. [18]	11 (implant removal, reoperation)	32 (revision, supplemental fixation, implant removal, reoperation)
	Sasso et al. [19]	10 (index/other level reoperation)	15 (index reoperation, bone stimulator, other cervical levels)
	Coric et al. [20]	6 (superficial infection, dysphagia/dysphonia)	14 (superficial infection, dural tear, dysphagia/dysphonia)
	Jawahar et al. [21]	N/A	N/A
	Our study	0	1
Adjacent segment disease	Our study	Managed conservatively: 0; revision advised/performed: 0	Managed conservatively: 2; revision advised/performed: 5 (13.88%)
Persistent neck pain	Our study	Absent: 36, present: 1 (2.7%)	Absent: 31, present: 5 (13.8%)
Trapezius pain	Our study	Absent: 34, present: 1 (2.1%)	Absent: 33, present: 3 (7.32%)

This study has several limitations that should be acknowledged. Its retrospective, nonrandomized design introduces potential selection bias, as surgical decision-making was influenced by patient preference, financial considerations, and surgeon discretion. The relatively small sample size compared with large multicenter randomized trials may limit statistical power and generalizability. An important limitation is the unequal follow-up duration between groups; the longer mean follow-up in the ACDF cohort reflects earlier adoption of fusion techniques prior to routine use of cervical disc arthroplasty and may overestimate the incidence of adjacent segment degeneration, disease, and reoperation rates, which should be considered when interpreting long-term outcomes. The use of the ODI instead of the NDI may limit direct comparison with other cervical spine studies, and potential confounders such as comorbidities, activity level, and occupational demands were not systematically controlled. Additionally, the lack of blinded outcome assessment and reliance on radiographic measurements subject to interobserver variability may affect the objectivity of certain findings. Patients who developed advanced heterotopic ossification after TDR represent a potential source of posttreatment selection bias. Heterotopic ossification is a known complication of cervical arthroplasty and may significantly influence postoperative ROM and adjacent segment biomechanics. Consequently, the findings related to motion preservation and adjacent segment kinematics should be interpreted within the context of patients maintaining functional arthroplasty and may not be generalizable to all TDR recipients. A notable limitation of this study is the sex imbalance between cohorts, with far fewer females in the ACDF group. This may have introduced confounding, as sex differences can affect pain perception, functional outcomes, and recovery. Future studies should aim for balanced sex distribution or adjust analyses to account for sex-related differences. Despite these limitations, strict inclusion criteria, age matching, and long-term follow-up enhance the internal validity and clinical relevance of this comparative analysis.

## Conclusions

TDR and ACDF both demonstrate comparable outcomes in terms of alleviating neck pain, radicular arm pain, and disability secondary to cervical degenerative disc disease when compared to ACDF with a minimum follow-up period of five years. While both procedures offer comparable improvements in myelopathic symptoms, TDR provides additional biomechanical advantages by more effectively preserving cervical spine alignment, kinematics at the index level, and at adjacent levels. This preservation of motion is associated with a reduced incidence of ASDeg and ASDis, which are known long-term complications of fusion procedures. Therefore, in appropriately selected patients, TDR represents an effective motion-preserving alternative to ACDF, with potential long-term biomechanical advantages. These findings should be interpreted in light of the unequal follow-up duration between the two cohorts.
